# Efficacy and Safety Outcomes of Intravitreal Dexamethasone Implant Therapy for the Treatment of Adult Coats' Disease

**DOI:** 10.1155/2020/9131908

**Published:** 2020-10-01

**Authors:** Qingshan Chen, Saying Liang, Xizhen Wang, Chenli Hu, Jieting She, Zi Li

**Affiliations:** Department of Ophthalmology, Shenzhen Eye Disease Prevention & Treatment Institute, Shenzhen Eye Hospital Affiliated to Jinan University, Shenzhen, China

## Abstract

**Purpose:**

To evaluate the efficacy and safety outcomes of dexamethasone intravitreal implant in patients with Stage 3A Coats' disease.

**Methods:**

A consecutive case series of adult Coats' disease managed with or without intravitreal dexamethasone implant (Ozurdex®, Allergan Inc., Irvine, California, USA) injection was retrospectively evaluated. The medical records of all included patients with a minimum follow-up of 6 months were reviewed. The patients were divided into two groups according to the application of dexamethasone implant as a DEX (+) group and DEX (−) group. Laser photocoagulation, anti-VEGF agents, and vitrectomy were performed if necessary. The primary outcomes included best-corrected visual acuity (BCVA), central retinal thickness (CRT), and intraocular pressure (IOP) at month 6. Resolution of the exudative retinal detachment (ERD), subretinal fluid (SRF), and vitreous hemorrhage (VH) was also collected.

**Results:**

Ten eyes (10 patients) with Stage 3A Coats' disease were included, and the mean follow-up time was 9.70 ± 4.42 months. The mean age was 44.20 ± 7.42 years, and 80% were male. Six eyes (6 patients) received intravitreal injection of Ozurdex were included in the DEX (+) group, while the other 4 eyes in the DEX (−) group. No significant difference of baseline characteristics including BCVA, CRT, IOP, and follow-up time can be defined between DEX (+) and DEX (−) groups. For the patients in the DEX (+) group, a significant improvement of BCVA was observed from the baseline of 1.28 ± 0.58 to 0.84 ± 0.66 logMAR at month 6 (*P*=0.03), while the CRT decreased from 970.33 ± 696.49 to 421.00 ± 275.76 *μ*m (*P*=0.067). For the DEX(−) group, BCVA changed from 0.76 ± 0.74 to 0.96 ± 0.60 logMAR at month 6 (*P*=0.066), while the CRT from 382.75 ± 17.68 to 412.75 ± 195.53 *μ*m (*P*=0.525) with no significant difference. IOP was elevated from 13.15 ± 1.74 mmHg at baseline to 18.05 ± 3.57 mmHg at month 6 with a *P* value of 0.02 for the DEX(+) group and from 14.48 ± 1.70 to 18.83 ± 4.06 mmHg (*P*=0.076) for the DEX (−) group. After a mean follow-up of 9.70 months, 5/6 (83.3%) eyes in the DEX (+) group and ¼ (25%) eye in the DEX (−) group achieved reattachment of ERD.

**Conclusion:**

Intravitreal dexamethasone implant therapy is effective for adult Stage 3A Coats' disease, which provides a new treatment option for ophthalmologists.

## 1. Introduction

Coats' disease is an idiopathic and nonhereditary condition caused by defect of outer retinal vascular development, characterized as retinal telangiectasia, with intraretinal and/or subretinal exudation, and without appreciable retinal or vitreous traction [1]. Coats's disease can be a serious threat to visual acuity when exudative retinal detachment (ERD), neovascular glaucoma (NVG), or other complications emerge as the progress of disease [2].

Coats' disease can affect both pediatric and adult population with a strong male predominance with no racial differences. The mean age at diagnosis is 10, and most cases are sporadic. Without medical interventions, Coats' disease can lead to severe complications, blindness, or even ocular atrophy [3]. Generally, the progression of Coats' disease in adults is slow, with peripheral retinal or perimacular angiotelectasis, accompanied by focal lipid deposition and scattered aneurysmal hemorrhage. Despite the slow progression, visual acuity can decline rapidly in patients with exudative retinal detachment.

The treatment of Coats' disease varies according to different stages of disease. In cases of Stage 1 patients present retinal telangiectasia alone, only closely follow-up is required unless sight threat signs are observed. For patients with telangiectasias and exudation (Stage 2), laser photocoagulation and cryotherapy treatment could be used for control of disease. If disease continuously progresses to Stage 3, causing the present of subtotal (Stage 3A) or total (Stage 3B) ERD, vitrectomy surgery combined with laser photocoagulation would be required. Due to the massive exudation characteristic of this stage, patients may undergo multiply resurgence and retreatment. For patients with Stage 4 characterized by the present of neovascular glaucoma (NVG), ocular enucleation is considered as a choice for whom suffered from unbearable pain causing by NVG, and Stage 5 is the terminal stage of Coats' disease, marked by a blind eye with cataract and phthisis bulbi [4].

Jun and Böhm et al. reported resolution of severe macular edema and remission of retinal telangiectasia and exudation in adult Coats' disease with intravitreal triamcinolone and bevacizumab injection [5]. Another previous study suggested that intravitreal antivascular endothelial growth factor (VEGF) agents can be considered as adjuncts to the traditional ablative treatment, with evident effect on resolution of macular edema and exudates secondary to Coats' disease [6]. Intravitreal triamcinolone or dexamethasone implant (Ozurdex) may be employed to improve anatomic and visual outcome in cases with a significant macular edema and subretinal fluid or exudates [7]. The current study aims to provide an updated report on the efficacy and safety outcomes of intravitreal dexamethasone implant therapy for adult Stage 3A Coats' disease. To the best of our knowledge, it is the first case series evaluating dexamethasone intravitreal implant in adult Coats' disease.

## 2. Methods

### 2.1. Study Design

This is a retrospective, open-label, nonrandomized case series conducted on adult patients (older than 18 years) with Stage 3A Coats' disease treated with Ozurdex or anti-VEGF agent combined with laser photocoagulation for macular edema and retinal nonperfusion with a mean follow-up period longer than 6 months. The study was designed in accordance with the tenets of the Declaration of Helsinki. Approval for the study protocol of this retrospective case series was obtained from the Research Ethics Board of Shenzhen Eye Hospital Affiliated to Jinan University, Shenzhen Eye Disease Prevention and Treatment Institute. Written informed consent was obtained from each patient before receiving the intravitreal injection, laser photocoagulation, and vitrectomy.

### 2.2. Participants

**Figure 1:**
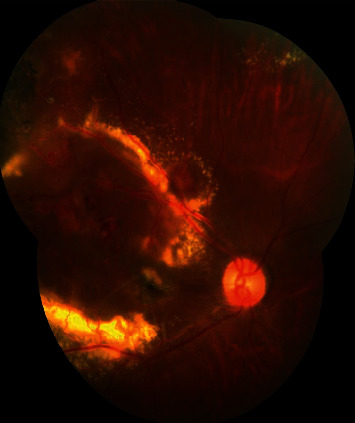
Color fundus photograph of a 40-year-old male patient (Case 4) in the DEX (+) group. The figure shows the right eye diagnosed with Coats' disease classified as Stage 3A. The baseline visual acuity of this patient was FC/30 cm. Retinal telangiectasia, microaneurysms, massive hard exudates, and exudative retinal detachment can be observed at the posterior pole.

**Figure 2:**
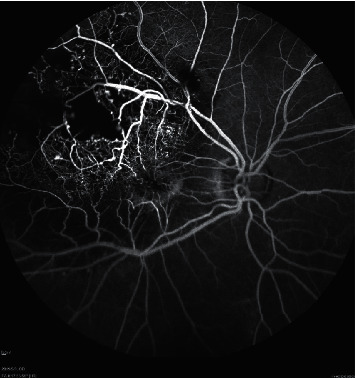
Fluorescein angiographic image of a 40-year-old male patient (Case 4) in the DEX (+) group. The figure shows the right eye diagnosed with Coats' disease classified as Stage 3A. The baseline visual acuity of this patient was FC/30 cm. Parafoveal and temporal retinal telangiectasia, aneurysmal vascular channels, and large area of capillary nonperfusion in the temporal retina can be observed at this early-phase fluorescein angiographic image.

Patients with Stage 3A Coats' disease who were treated at Shenzhen Eye Hospital Affiliated to Jinan University were retrospectively included in our study, as defined by retinal telangiectasia with massive intraretinal or subretinal exudation, and subtotal ERD (shown in Figure 1). Ophthalmologic examinations including fundus photograph, B-scan ultrasonography, fundus fluorescein angiography (FFA) (Spectralis HRA, Heidelberg Engineering, Heidelberg) (shown in Figure 2), and optical coherence tomography (OCT) (Cirrus HD OCT-5000, Zeiss, Jena, Germany) were performed in all patients. The exclusion criteria were as follows: (i) patients presenting iris neovascularization or anterior chamber cholesterolosis and (ii) patients having other uncontrolled systemic diseases and (iii) vision acuity lower than finger count for the target eye.

### 2.3. Treatments

All included cases underwent laser photocoagulation or anti-VEGF injection as necessary. Among them, 6 cases received intravitreal injection of Ozurdex during the follow-up period were marked as the DEX (+) group, and the other 4 cases did not receive Ozurdex treatment were marked as the DEX (−) group. Additional treatments including laser photocoagulation, anti-VEGF treatment, and vitrectomy will be applied if necessary depending on the researchers' judgement.

Intravitreal injections were performed in the operation room under topical anesthesia. Ozurdex (0.7 mg DEX intravitreal implant) and 0.05 ml (0.5 mg) conbercept (Sichuan Kanghong Biotechnology Ltd., China) were injected into the vitreous cavity through the pars plana at 3.5 mm posterior to the limbus. Indirect ophthalmoscopy was performed immediately after the injection in order to confirm the presence of implant in vitreous cavity and normal central retinal artery perfusion. Laser photocoagulation was performed with a 577 nm wavelength laser (Quantel Medical, Cedex, France) on retinal nonperfusion area.

### 2.4. Data and Analysis

Information collected included details of the underlying diseases, best-corrected visual acuity (BCVA), treatments, and pre- and post-treatment central retinal thickness (CRT) measured by spectral domain optical coherence tomography (SD-OCT), intraocular pressure (IOP), retinal status, and other documented complications. BCVA was converted into 10 logarithms of the minimum angle of resolution letters (logMAR). For the cases with visual acuity worse than 5/200, the following conversion was used: counting fingers = 1.85 logMAR and hand movements = 2.3 logMAR according to a previous study [8]. All data are presented as mean ± standard deviation. Statistical analyses were performed between the two groups with/without DEX treatment on the BCVA, CRT, and IOP data using paired sample t-tests with SPSS Statistics 26.0 (Statistical Analysis System Institute Inc., Cary, N.C.). A *P* value of less than 0.05 was considered statistically significant.

## 3. Results

### 3.1. Patients

Ten patients (10 eyes) with Stage 3A Coats' disease were included in the present retrospective study. There were 8 males (80%) and 2 females (20%) with a mean age of 44.2 ± 7.42 years (range, 34–59 years). The mean follow-up time was 9.7 ± 4.42 months (range, 6–18 months). In this study, all studied eyes had a pretreatment VA of ≤0.2logMAR, and macular edema was confirmed in all included eyes by optical coherence tomography (OCT) and fundus fluorescein angiography (FFA). The baseline clinical characteristics are summarized in Tables 1 and 2. The baseline BCVA for the DEX (+) group was 1.28 ± 0.58 while that of the DEX (−) group was 0.76 ± 0.74 (*P*=0.247). The baseline CRT of the DEX (+) group and DEX (−) group was 970.33 ± 696.49 *μ*m and 382.75 ± 171.75 *μ*m, respectively (*P*=0.096). No significant of IOP can be observed between the two groups (13.15 ± 1.74 mmHg vs. 14.48 ± 1.70 mmHg, *P*=0.269) with a similar length of follow-up period (10.67 ± 5.47 months vs. 8.25 ± 2.06 months, *P*=0.359). Treatment and retinal outcome details during the study period are summarized in Table 3.

### 3.2. Macular Edema and CRT

**Figure 3:**
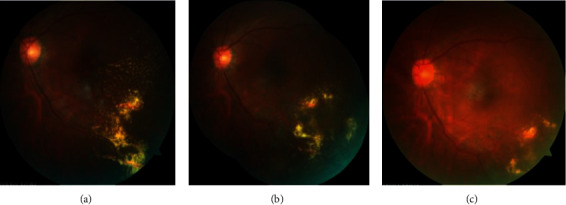
Color fundus photograph of Case 1 in the DEX (+) group before and after the intravitreal injection of DEX implant, conbercept, and laser treatment. The patient was diagnosed with Coats' disease classified as Stage 3A and consecutively observed for 16 months. 3A: color fundus photograph showing temporal periphery and macular telangiectasia, microaneurysms, large hard exudates, and ERD before intravitreal injection DEX implant, conbercept, and laser treatment; 3B: macular edema and lipid hard exudation were absorbed 4 months after the treatment, while the laser scar can be observed at temporal peripheral retina. 3C: retinal lipid hard exudation was absorbed absolutely at the macular area and temporal peripheral retina 1 year after the treatment.

**Figure 4:**
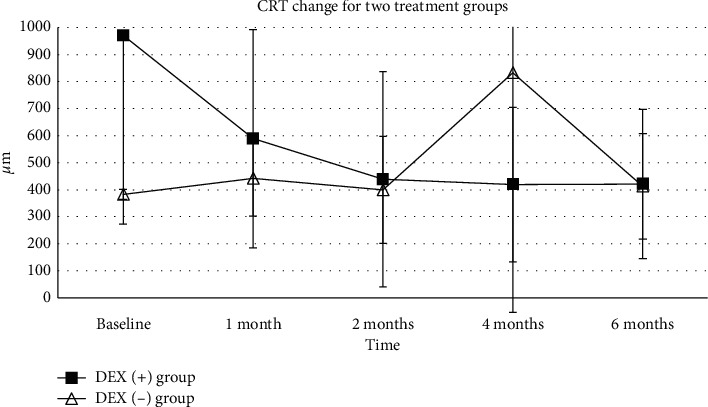
Central retinal thickness (CRT) change for the DEX (+) group and DEX (−) group. The CRT at the baseline and 1, 2, 4, and 6 months during the follow-up period is shown in this figure. The change of CRT for the DEX (+) group was defined with a line chart with solid square, while the change of CRT for the DEX (−) group was defined with a line chart with open arrowhead (^*∗*^*P* < 0.5, ^*∗∗*^*P* < 0.1, and ^*∗∗∗*^*P* < 0.01).

The mean baseline CRT for all patients (*n* = 10) was 735.3 ± 609.4 *μ*m. Although all studied eyes showed a certain degree of decreasing CRT on SD-OCT, no significant difference was detected with a *P* value of 0.09 (the paired samples t-test) at month 6. The intergroup analysis revealed no statistically significant difference between the DEX (+) group and DEX (−) group. Macular edema and lipid exudate were found absorbed in Case 1 in the DEX (+) group 4 months after initial intravitreal implant of Ozurdex (shown in Figure 3). For Case 9 in the DEX (−) group, total ERD associated with disease progression with a CRT of 2128 *μ*m occurred and underwent a vitrectomy surgery. However, ERD was still found after surgical operation. Persist macular edema was observed in 2 eyes in the DEX (+) group and 2 eyes in the DEX (−) group. As the poor anatomic prognosis due to submacular exudate, additional treatment was performed for those patients. The change of central retinal thickness for two groups is shown in Figure 4. For the DEX(+) group, the CRT decreased from 970.33 ± 696.49 *μ*m at baseline to 588.67 ± 402.78, 438.67 ± 397.86, 419.67 ± 285.41, and 421.00 ± 275.76 *μ*m at month 1, 2, 4, and 6 (*P*=0.073, 0.063, 0.056, and 0.067).

### 3.3. Visual Acuity

**Figure 5:**
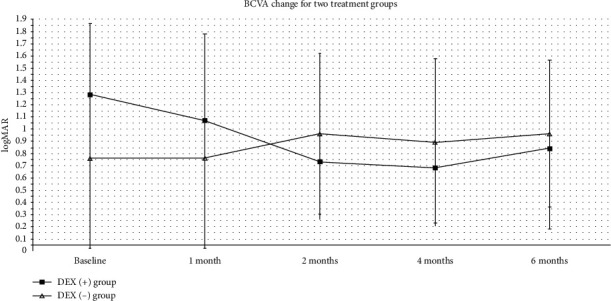
Best-corrected visual acuity (BCVA) change for the DEX (+) group and DEX (−) group. The BCVA at the baseline and 1, 2, 4, and 6 months during the follow-up period is shown in this figure. The change of BCVA for the DEX (+) group was defined with a line chart with solid square, while the change of BCVA for the DEX (−) group was defined with a line chart with open arrowhead (^*∗*^*P* < 0.5, ^*∗∗*^*P* < 0.1, and ^*∗∗∗*^*P* < 0.01).

The mean baseline BCVA for all treated eyes was 1.08 ± 0.67 logMAR, which was observed as 0.89 ± 0.61 logMAR at month 6 with no significant change (*P*=0.21, paired sample t-test). No statistical difference between the two groups was defined during the follow-up time. Subgroup analysis revealed that there is a statistically significant improvement at 2, 4, and 6 months after initial treatment in the DEX (+) group with a *P* value of 0.002, 0.007, and 0.03, respectively. Meanwhile, there was no statistically significant difference for BCVA improvement discovered in the DEX (−) group during the entire follow-up. The change of BCVA for two groups is shown in Figure 5.

### 3.4. Intraocular Pressure

**Figure 6:**
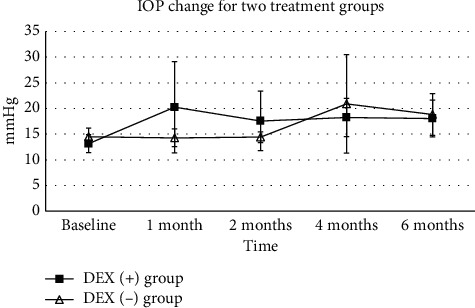
Intraocular pressure (IOP) change for the DEX (+) group and DEX (−) group. The IOP at the baseline and 1, 2, 4, and 6 months during the follow-up period is shown in this figure. The change of IOP for the DEX(+) group was defined with a line chart with solid square, while the change of IOP for the DEX(-) group was defined with a line chart with open arrowhead (^*∗*^*P* < 0.5, ^*∗∗*^*P* < 0.1, and ^*∗∗∗*^*P* < 0.01).

Increasing IOP was the only treatment-related adverse event observed. The mean baseline IOP was 13.68 ± 1.77 mmHg for all studied eyes and elevated to 18.36 ± 3.57 mmHg with a statistically significant difference (*P*=0.001). Among all patients, 30% had IOP higher than 25 mmHg of which 2 cases had IOP above 35 mmHg at 1 or more visits during the follow-up period. For patients in the DEX (+) group, IOP moderately rose in a temporary fashion, and all patients managed with IOP lowering medication. One case in the DEX (−) group had an elevating IOP above 35 mmHg due to total ERD and reduced to 24 mmHg at month 6. No glaucoma laser surgery or glaucoma incisional surgery during the follow-up period was required. No statistical difference of IOP change was defined between the two groups (shown in Figure 6).

## 4. Discussion

Among the 10 Stage 3A Coats' disease patients in the present study, 2 cases (20%) had visual acuity of finger counting because of ERD or persistent macular subretinal scar, and 3 cases (30%) had visual acuity better than 0.5 logMAR after 6 months of follow-up. For the baseline characteristics, no significant difference can be defined between DEX (+) and DEX (−) groups including BCVA, CRT, IOP, and follow-up time. One case in the DEX (−) group progressed to total ERD and underwent vitrectomy surgery. For the rest cases, no one advanced to more severe stage.

It has been verified that impairment of the blood-retinal barrier can lead to intraretinal and subretinal fluid (SRF), and blood and lipid accumulation in advanced stages of Coats' disease (Stage 3A and 3B), resulting in massive retinal exudation or serous retinal detachment [9]. Laser photocoagulation or cryotherapy is often ineffective in these stages. With the emerging of anti-VEGF agents, several studies have revealed the promising efficacy in decreasing the amount of SRF, macular edema, and exudates of bevacizumab and ranibizumab in Stages 3 and 4 Coats' disease [10–12]. However, it may take repetitious injections to suppress the disease progression since a great number of studies have shown that aqueous and vitreous levels of VEGF in Coats' disease are approximately 1000 pg/ml, which is much higher than that in patients with choroidal neovascular membranes secondary to age related degeneration [13, 14]. However, there is a drawback for anti-VEGF agents due to the unavoidable risk of tractional retinal detachment has been founded in previous studies [12, 15].

Periocular or intravitreal injection of corticosteroids for Coats' disease has shown its effect on attenuate leukostasis and vascular leakage along with suppression of ocular inflammation before [16, 17]. However, the poor safety profile of triamcinolone acetonide has limited its application; therefore, dexamethasone implant can play a better role in this complex disorder with its long duration and acceptable safety profile.

Several studies have indicated the efficacy and safety of dexamethasone intravitreal implant in the initial management of Coats' disease [7, 18]. Martínez-Castillo et al. firstly reported a 46-year-old female with Coats' disease who was successfully managed with the dexamethasone intravitreal implant (Ozurdex®) combined with retinal photocoagulation. The patient showed no further recurrence of ERD, and the extensive lipid exudation progressively regressed after 1 year follow-up [18]. Saatci et al. demonstrated similar results in 2 pediatric patients with Coats' disease. In this study, intravitreal Ozurdex was effective to complete resolution of ERD in a Stage 3A case which in turn rendered the retina amenable to laser photocoagulation. Thus, Ozurdex was made as an adjuvant therapy in addition to laser photocoagulation or intravitreal anti-VEGF agents [7]. Kumar et al. revealed an adult-onset Coats' disease concomitant with retinal vasoproliferative tumor (VRT) which received intravitreal Ozurdex due to massive exudation and hemorrhages in the temporal peripheral retina, and ERD. Retinal photocoagulation was performed 1 month after intravitreal Ozurdex, and after 4-month initial treatment, retinal extensive lipid accumulation progressively regressed with a stable vision of 20/60 and resolved macular edema, while no adverse effect of steroid on IOP or the lens was observed during the follow-up period. This study suggested that intravitreal dexamethasone implant (Ozurdex) may be an effective initial therapeutic approach for Coats' disease with massive exudation [19].

**Figure 7:**
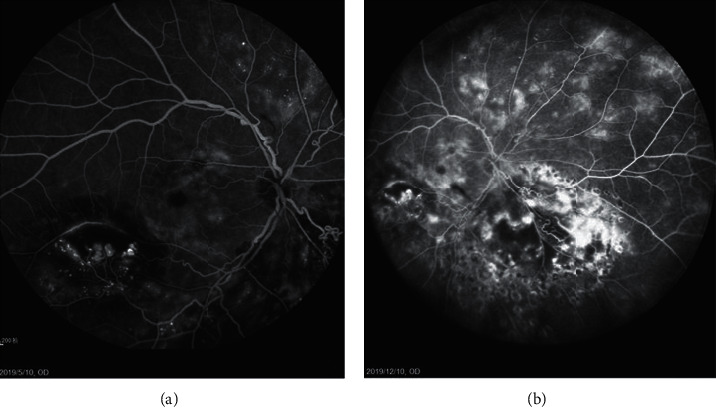
Fluorescein angiographic image of Case 8 in the DEX (−) group before and after the laser photocoagulation, intravitreal injection of conbercept (IVC), and transpupillary thermotherapy (TTT). 7A: fluorescein angiographic image showing the retinal telangiectasia, large aneurysmal vascular channels, macular ERD, and periphery capillary nonperfusion at inferior, temporal, and nasal retina before treatment; 7B: fluorescein angiographic image revealed retinal telangiectasia, persist macular ERD, periphery capillary nonperfusion, and leakage at inferior, temporal, and nasal retina after treatment.

The present study is the first case series evaluating the efficacy and safety outcomes with or without intravitreal dexamethasone implant therapy in adult Stage 3A Coats' disease. For the DEX (+) group, retinal laser photocoagulation combined with Ozurdex results in an almost significant decrease of CRT (*P*=0.067) with a notable improvement in BCVA after 6-month follow-up. Although the *P* value is a bit bigger than 0.05, it may be associated with the relatively small scale of the present study and suggests a bigger scale study in the future. During the study period, 4 patients required additional treatment because of relapse of macular edema or subretinal hemorrhage accumulation, 3 cases accepted intravitreal injection of conbercept, and 1 proceeded with a second intravitreal Ozurdex. At the endpoint of the study, 4 patients showed a reattachment of exudative RD and abatement of retinal exudation, while in the DEX (−) group, 4 patients were treated with a combination therapy of laser photocoagulation and intravitreal injection of conbercept. At the endpoint of our study, 3 cases in the DEX (−) group showed persistence ERD (shown in Figure 7), including 1 case progressed to Stage 3B with a macular hole three months after intravitreal injection, and a 25-G pars plana vitrectomy (PPV) was performed to relocate and repair the retina. Another patient received TTT because of temporal and nasal capillary telangiectasia. In order to avoid the confusion, no DEX was used for the patients in the DEX (−) group. And at the endpoint of follow-up, only 1 case achieved reattachment retina with reduced lipid exudation in the DEX (−) group. Although there was no significant difference between the two groups both for visual acuity and anatomical outcomes, it cannot be denied that intravitreal dexamethasone implant could be a valuable addition to the treatment options for Coats' disease, as it has demonstrated an advantageous efficacy on decreasing macular edema and promoted the absorption of exudation and hemorrhages.

The pathological processes in Coats' disease include the breakdown of the blood-retinal barrier, which leads to the thickening of vascular endothelium and causes fluid and lipid exudation accumulates within the retina. Coupled with the presence of aberrant pericytes and endothelial cells, leading to the weakening of the retinal vessel walls, thus result in the formation of telangiectasis, aneurysms, and progressive leakage, as well as vessel closure, which leads to ischemia [4]. Therefore, it is crucial for Stage 3 patients to receive prompt treatment for reducing retinal exudation and to facilitate subsequent laser photocoagulation to prevent the disease progressing, since Stage 3 is at the cusp of progressing to ERD or NVG. Near yellow wavelength has better absorbed by the blood in the target vascular channels, so we chose a 577 nm yellow laser as initial laser photocoagulation. In our study, we had not performed cryotherapy because all the lesions are located in the posterior pole.

There are several limitations in our study that must be mentioned. First, it is a retrospective study and not randomized. The eyes receive Ozurdex or not for adult Coats' disease were collected. Second, numbers of the included patients in both groups are relatively small, leading to lower strength of convincing. Undoubtedly, prospective studies with a larger sample size are required and more cases remain to be collected.

In conclusion, intravitreal dexamethasone implant therapy (Ozurdex®) provides a potential candidate as corticosteroids, which are well known for inhibiting retinal inflammation, repairing the tight junctions between vascular endothelial cells [20], for the treatment of Coats' disease. Our study demonstrated a promising efficacy of intravitreal Ozurdex in adult Coats' disease with decreasing macular edema and exudation, and noticeable visual acuity improvement with reattached ERD. Further studies with a larger sample are required to confirm its efficacy and safety and make further conclusion.

## Figures and Tables

**Table 1:** Baseline clinical characteristics between two groups.

	DEX (+)	DEX (−)	*P*
BCVA (logMAR), mean ± SD	1.28 ± 0.58	0.76 ± 0.74	0.247
CRT (*μ*m), mean ± SD	970.33 ± 696.49	382.75 ± 171.75	0.096
IOP (mmHg), mean ± SD	13.15 ± 1.74	14.48 ± 1.70	0.269
Follow-up (months), mean ± SD	10.67 ± 5.47	8.25 ± 2.06	0.359

BCVA: best-corrected visual acuity. logMAR: 10 logarithm of minimum angle of resolution letters. CRT: central retinal thickness. IOP: intraocular pressure. DEX: dexamethasone intravitreal implant.

**Table 2:** Baseline clinical characteristics of patients with Stage 3A Coats' disease.

Case no.	Age/gender	Systemic disease	Prior treatment	BCVA (logMAR)	CRT (*μ*m)	IOP (mmHg)	Retinal details at first visit
Case 1	41/male	Fatty liver disease	Intravitreal ganciclovir	1.0	552	12.8	Exudative retinal detachment
Case 2	52/male	None	IVC, laser	1.4	228	10	Subtotal ERD with massive exudates
Case 3	59/male	None	None	1.85	1492	13.9	Subtotal ERD with massive exudates and epiretinal membrane
Case 4	40/male	None	Laser	1.85	2111	13	Subtotal ERD with subretinal hemorrhage
Case 5	45/male	None	IVR	1.3	732	14.2	Extensive peripheral retinal vascular telangiectasia
Case 6	47/female	None	None	0.3	707	15	Subtotal ERD with massive exudates
Case 7	36/male	None	None	0.5	640	16	Considerable exudation involving the posterior pole
Case 8	46/female	None	None	0.2	291	13	Subtotal ERD with massive exudates
Case 9	34/male	None	IVC, laser	1.85	290	13	Subtotal ERD with massive exudates
Case 10	42/male	Diabetic	None	0.5	310	15.9	Subtotal ERD with massive exudates

BCVA: best-corrected visual acuity. logMAR: 10 logarithm of minimum angle of resolution letters. CRT: central retinal thickness. IOP: intraocular pressure. DEX: dexamethasone intravitreal implant. IVC: intravitreal injection of conbercept. PPV: pars plana vitrectomy. ERD: exudative retinal detachment.

**Table 3:** Treatments and retinal outcome details of patients with Stage 3A Coats' disease.

Case no.	Baseline BCVA (logMAR)	Initial treatments	Additional treatment	Reason for additional treatment	Time to additional treatment	Following time (months)	Final BCVA (logMAR)	Final retinal outcome
Case 1	1	DEX, VC, and laser	—		—	16	0.1	Reattachment of ERD, lipid exudative absorbed
Case 2	1.398	DEX, followed by laser after 1 month	Anti-VEGF	Retinal haemorrhage, leakage of retinal telangiectasia	3 months	6	1.0	Reattachment of ERD, lipid exudative absorbed
Case 3	FC/30 cm	Laser, DEX	—		—	12	1.85	Macular subretinal scar
Case 4	FC/30 cm	Laser, DEX	Anti-VEGF	Macular ERD, neovascularization	1 week	6	1.0	Persistence ERD
Case 5	1.301	Laser, DEX	DEX	Recurrent macular ERD	10 months	18	1.0	Reattachment of ERD, lipid exudative absorbed
Case 6	0.301	Laser, DEX	Anti-VEGF	Macular ERD	1 week	6	0.1	Reattachment of ERD, lipid exudative absorbed
Case7	0.523	Laser, IVC	—		—	8	0.8	Persistence ERD
Case 8	0.523	Laser, IVC	TTT	Temporal and nasal side capillary telangiectasia	8 months	8	0.5	Persistence ERD
Case 9	1.85	Laser, IVC	PPV	Extensive posterior pole ERD, macular hole	4 months	6	1.85	Persistence ERD
Case 10	0.5	Laser, IVC	—		—	11	0.7	Reattachment of ERD, lipid exudative reduced

BCVA: best-corrected visual acuity. logMAR: 10 logarithm of minimum angle of resolution letters. CRT: central retinal thickness. IOP: intraocular pressure. DEX: dexamethasone intravitreal implant. IVC: intravitreal injection of conbercept. PPV: pars plana vitrectomy. ERD: exudative retinal detachment. TTT: transpupillary thermotherapy.

## Data Availability

The data used to support the findings of this study are available from the corresponding author upon request.
